# Methods for detecting methylation by SNP interaction in GAW20 simulation

**DOI:** 10.1186/s12919-018-0140-y

**Published:** 2018-09-17

**Authors:** E. Warwick Daw, James Hicks, Petra Lenzini, Shiow J. Lin, Judy Wang, Christine Williams, Ping An, Michael A. Province, Aldi T. Kraja

**Affiliations:** Division of Statistical Genomics, Department of Genetics, Center for Genome Sciences and Systems Biology, Washington University School of Medicine, 660 Euclid Ave., Saint Louis, MO 63110 USA

## Abstract

To examine whether single-nucleotide polymorphism (SNP) by methylation interactions can be detected, we analyzed GAW20 simulated triglycerides at visits 3 and 4 against baseline (visits 1 and 2) under 4 general linear models and 2 tree-based models in 200 replications of a sample of 680 individuals. Effects for SNPs, methylation cytosine-phosphate-guanine (CpG) effects, and interactions for SNP/CpG pairs were included. Causative SNPs/CpG pairs distributed on autosomal chromosomes 1 to 20 were tested to examine sensitivity. We also tested noncausative SNP/CpG pairs on chromosomes 21 and 22 to estimate the empirical null. We found reasonable power to detect the main causative loci, with the exact power depending on sample size and strength of effects at the SNP and CpG sites.

## Background

### Introduction

DNA methylation is an important epigenetic mark at transcriptional start sites, regulatory elements, repeat sequences, or within a gene [1]. Methylation’s main effect is to silence genes, which are dynamically regulated in expression. Methylation/demethylation confers genome stability, gene expression control, and contributions in biological functions and development [2].

We analyzed GAW20 simulated methylation and triglyceride (TG) levels at time points before and after “genomethate” treatment. We examined Type I error and power for identifying single-nucleotide polymorphisms (SNPs) by methylation interactions under several methods with different models. We conducted analyses with prior knowledge of solutions to the GAW20 problem. Our goal was to examine the feasibility of detecting gene by methylation interactions.

### Data

The GAW20 simulated data contains 200 replicates, each with SNP by methylation interaction effects at 5 main effect SNP/CpG (cytosine-phosphate-guanine) pairs and 100 very-small-effect “background” SNP sites. All simulated causative SNPs were distributed on chromosomes 1 to 20. Chromosomes 21 and 22 were left without any simulated effects, so they could be used for tests under the null distribution. We identified 2267 SNPs with nearby methylation markers on chromosomes 21 and 22, which we used to generate empirical null distributions for identifying SNP by methylation interactions. For full details of the GAW20 simulation, please refer to the data description paper Aslibekyan S, et al. (2017) [3].

## Methods

We focused on 4 methods. For the first 2 methods, we developed a set of overlapping models:

*Model 1a: Post* = *β*_0_ + *β*_1_*SNP* + *β*_2_*CpG* + *β*_3_*SNP* ∗ *CpG* + *β*_*C*_ + *ε*.

This is the generating model for the simulation: it includes SNP main effect, CpG methylation (expressed as (1 − CpG) because less methylation results in more expression) and their interaction effect on posttreatment TGs. *Post* is the average of log (TG3) and log (TG4); *β*_0_ is the intercept; *β*_1_*SNP* is the SNP effect; *β*_2_*CpG* is the methylation effect; *β*_3_*SNP* ∗ *CpG* is their interaction effect; *β*_*C*_ corresponds to a vector of covariates [age, age^2^, sex, and the average of log(TG1) and log(TG2) before “genomethate” treatment (*Pre*)]; and *ε* is the residual.

*Model 1b: Post* = *β*_0_ + *β*_1_*SNP* ∗ *CpG* + *β*_*C*_ + *ε*

This is a reduced version of model 1a that includes a SNP-by-methylation interaction, but no SNP or methylation main effects. Because there are strong interaction effects at the 5 main simulated SNP-by-CpG effect sites, we wanted to test whether this model had better power because of having fewer terms.

*Model 2a*: *δ*_*TG*_ = *β*_0_ + *β*_1_*SNP* + *β*_2_*δ*_*CpG* + *β*_3_*SNP*^∗^ *δ*_*CpG* + *β*_*C*_ + *ε*

This model represents a “reasonable” model that is not the generating model. Because in practice the “true” model is unknown, we also applied a model differing from the generating model. Included are the main effect of the SNP, the main effect *δ*_*CpG* (methylation change pre- to posttreatment), and their interaction effect on the *δ*_*TG*_ pre- to posttreatment [the change of average log (TG) at times 3 and 4 versus average of log (TG) at times 1 and 2]. Here *δ*_*TG*_ is change of TG (*Post* – *Pre*); *δ*_*CpG* is the methylation difference between visits 4 and 2; and *β*_*C*_ is the beta coefficient for the covariates (age, age^2^, and sex).

*Model 2b*: *δ*_*TG*_ = *β*_0_ + *β*_1_*SNP*^∗^ *δ*_*CpG* + *β*_*C*_ + *ε*

This is a reduced version of model 2a, in which only the SNP by *δ*_*CpG* methylation interaction effect is included. The rationale for this model was the same as with model 1b.

We applied the following 4 methods.

- Method I: General linear models

We used Proc GLM in SAS for fitting linear models by the least squares approach, and applied it to models 1a and 1b [4]. In this model, we accounted for the family data by including pedigree ID as a random effect, which can provide a sufficient method to account for the primarily nuclear families found here.

- Method II: Mixed models

We used mixed models to adjust for relatedness and repeated measures by way of structured covariance models. The parameters were estimated by the likelihood technique using Proc MIXED in SAS, which was applied to models 1a, 1b, 2a, and 2b [5]. With this method, we included the family ID as a class variable and used it as a “subject” variable, effectively making it a random effect, similar to Method I.

- Method III: Regression trees

Tree-based models nonparametrically predict outcomes by partitioning data into bins based on rules learned from the data. We use these to predict *Post* (models 1a and 1b) using the 10 causative SNPs and CpGs. Because the sample size of each replication is relatively small (*n* = 680), we set the condition that in the final leaves a minimum number of 10 can be considered as a limit for a final split. These analyses were performed via SAS (v. 9.4) and SAS Data Mining (v. 14.1) [5, 6]. The regression tree analyses presented here were implemented at the full data level (200 replications combined), which created a more generalizable application case with similarity to the existing GWAS large consortia data.

- Method IV: Random forests

Random forests predict outcome variables by averaging the predictions of a large number of uncorrelated regression trees. This can allow for better model performance in data sets with smaller sample size or many predictors. For each replicate we created a forest of 10,000 trees to predict *Post* (models 1a and 1b) based on the pretreatment level, age, sex, causative and background SNPs, and methylation sites (113 predictors). We created this model using the *scikit-learn* software package [7].

In addition, heritabilities were estimated at 2 levels: at the full polygenic model using SOLAR (Sequential Oligogenic Linkage Analysis Routines) software [8] (http://solar-eclipse-genetics.org/) and at the single SNP level, in this case squaring beta coefficient for SNP (where using standardized dosages versus standardized residuals of a normally distributed response variable after adjusting for covariates. The b^2^ in this case provides an approximate estimate of additive effect) [9].

To obtain an empirical null distribution for Methods I and II, we identified 2267 SNP–CpG pairs on chromosomes 21 and 22, where there were no simulated causative SNPs. A pair of markers was identified by selecting a SNP, and a CpG that is adjacent but has a higher base-pair position than the SNP. For chromosome 21, we formed 817 pairs from 10,385 SNPs and 4157 CpG sites. For chromosome 22, we formed 1450 pairs from 9464 SNPs and 8381 CpG sites.

## Results

Testing of *Post* (used in models 1a and 1b) for 200 replications, indicated a heritability of 43% (Table 1). Examination of the traits distributions after log transformation suggested the normal error assumption is not unreasonable and use of linear regressive methods appropriate.

**Table 1:** Heritability estimates of trait *Post* (using SOLAR)

Trait/Marker(s)	Mean	Median	Standard Deviation	Software
*Post* [average of log (TG3) and log (TG4)]	0.4307	0.4313	0.0509	SOLAR
rs9661059	0.0210	0.0210	0.0050	b^2^ regression (SAS) from standardized dosage and standardized phenotype
rs736004	0.0008	0.0005	0.0008	-″-
rs1012116	0.0070	0.0070	0.0030	-″-
rs10828412	0.0004	0.0002	0.0005	-″-
rs4399565	0.0090	0.0080	0.0030	-″-
100 background SNPs	0.0024	0.0011	0.0032	b^2^ regression (SAS) from standardized dosage and standardized phenotype

Five main SNPs (β^2^ of a regression of standardized residuals on standardized dosage for each marker) and 100 background SNPs evaluated at 200 replications

The power to detect causative SNP by methylation interactions was modest to good with Methods I and II (Table 2). The full GLM model (model 1a and Method I) gave power ranging from 0.91 down to 0.20 with α = 0.05 for detecting the SNP by methylation interaction at the 5 primary causative sites (Table 2). The reduced GLM model, which we thought might perform better from having fewer terms, had lower power for some sites and higher power for others, depending on the strengths of the effects and the levels of methylation at each site. The very-small-effect “background” sites were slightly better detected by the reduced model (0.07 vs 0.06 for the full model), but power was very poor in both cases for these tiny, but real, effects. The full mixed model (model 1a and Method II) also showed modest to good power to detect causative SNP–CpG pairs: 0.95 down to 0.28 for the 5 main sites, and the power at each site was slightly better than for the full GLM model. The reduced mixed model (model 1b and Method II) showed better power at some sites and worse power at others, in the same directions as Method I. The detection of the “background” sites was best with the reduced mixed model, at 0.12, but in the full mixed model, this detection was no better than the null. In addition to the generating model, with the mixed model we also tested change between simulated log (TG) at times 3 and 4 versus times 1 and 2 with effects from methylation level change from time 4 versus time 2 (models 2a and 2b with Method II). We see a small loss of power for this full model (2a) vs. the full generating model (1a), with power from 0.86 to 0.29 at the 5 main sites for this model. However, there is a substantial loss of power in the reduced model (power ranging from 0.19 down to 0.05). This may suggest caution is required in applying the reduced model in situations where the mechanism is not well understood. In such situations, it may be prudent to apply the full model.

**Table 2:** Proportion of tests with *p* < 0.05 under several models with *proc GLM* and *proc MIXED*

	Number of tests	GLM model 1a	GLM model 1b	MIXED model 1a	MIXED model 1b	MIXED model 2a	MIXED model 2b
cg0000036 * rs9661059	200	0.91	0.41	0.95	0.64	0.86	0.11
cg0004591 * rs1082841	200	0.20	0.28	0.28	0.40	0.29	0.06
cg0124267 * rs4399565	200	0.52	0.62	0.59	0.72	0.44	0.19
cg1048095 * rs736004	200	0.63	0.18	0.69	0.28	0.66	0.05
cg1877239 * rs1012116	200	0.78	0.45	0.83	0.62	0.74	0.12
Background SNPs	20,000	0.06	0.07	0.05	0.12	0.05	0.04
Null SNP interactions	453,400	0.05	0.05	0.05	0.05	0.05	0.05
Null SNP interactions (w/MAC > 50)	380,800	0.05	0.05	0.05	0.05	0.04	0.04

The *p* values at for the “null” sites on chromosomes 21 and 22 were distributed more or less as expected with Type I error of 0.04 to 0.05, close to nominal α = 0.05, under Methods I and II (see Table 2). However, some caution should be noted here. In an initial run (data not shown), we inadvertently used the sandwich estimator, which produced inflated *p* values with low minor allele frequency SNPs.

Finally, we used regression tree models (Methods III and IV) as an alternative to linear models. Table 3 shows the results of these methods. Individual regression trees have good performance in scenarios with large sample sizes and fewer predictors. The regression tree results represent a tree trained on all 200 replications combined (*n* = 136,000). In the combined data, the major known simulated SNP and CpG markers had the highest importance values. Conversely, random forests show high performance in scenarios with more weak predictors, but not necessarily a large sample size. We tested the relative importance of the major predictors in a random forest model (Method IV) for each replicate, and included background SNPs as superfluous predictor variables. After covariates, the CpG sites reliably achieved the highest importance scores, while the SNPs that they modified had lower importance.

**Table 3:** Results from decision tree analysis on *Post*

	Decision tree (importance)		Random forest (importance rank)	
Predictor	Training	Validation	Mean (SD)	Median
rs736004	1.0000	0.9645	33.86 (27.75)	22
rs9661059	0.9989	1.0000	14.8 (13.29)	10
rs10828412	0.9843	0.9860	34.75 (24.16)	25.5
rs1012116	0.8998	0.8922	20.25 (15.08)	14
rs4399565	0.7838	0.6970	23.27 (15.25)	19
cg18772399	0.6793	0.5597	4.415 (1.72)	4
cg00000363	0.5758	0.4951	4.085 (1.75)	4
cg10480950	0.5348	0.3802	4.59 (1.91)	4
cg00045910	0.4441	0.3508	4.715 (1.74)	5
cg01242676	0.4333	0.2718	4.7 (1.79)	5
Pre	–	–	1 (0)	1
Age	–	–	5.075 (1.74)	5
Sex	–	–	87.625 (10.21)	90

Decision analysis consisted of all 200 replications combined, and then separated into training (*n* = 54,334), validation (*n* = 40,769), and test (*n* = 40,897). Random forests were performed for each replicate separately, and each variable was ranked for importance. *Pre, age,* and *sex* were included as covariates

## Discussion and conclusions

The heritability estimated reported suggested the simulated phenotypes had a good inheritance pattern, making it possible to detect causative SNPs and SNP–CpG interactions in our analyses. In examining the frequency of tests with *p* value of interactions ≤0.05 in 4 models with mixed model analysis (models 1a, 1b, 2a, and 2b for Method II; see Table 2) and 2 models with GLM analysis (models 1a and 1b for Method I; see Table 2), we see reasonable power to detect these effects in this sample. When the exact mechanism is unknown, the results from models 2a and 2b suggest it may be prudent to use a full model with both main and interaction terms, but when the mechanism is well understood, neither the full model (1a) nor the reduced model (1b) was always advantageous. Larger samples are required to detect these effects with these methods after correction for multiple testing. However, these results suggest that, given a sufficient sample, it is possible to detect gene by methylation interactions with the methods used here.
